# The Intestinal Microbiota and Colorectal Cancer

**DOI:** 10.3389/fimmu.2020.615056

**Published:** 2020-11-30

**Authors:** Yiwen Cheng, Zongxin Ling, Lanjuan Li

**Affiliations:** Collaborative Innovation Center for Diagnosis and Treatment of Infectious Diseases, State Key Laboratory for Diagnosis and Treatment of Infectious Diseases, National Clinical Research Center for Infectious Diseases, The First Affiliated Hospital, School of Medicine, Zhejiang University, Hangzhou, China

**Keywords:** biomarker, colorectal cancer, dietary intervention, intestinal microbiota, inflammation, metabolites

## Abstract

The intestinal microbiota, composed of a large population of microorganisms, is often considered a “forgotten organ” in human health and diseases. Increasing evidence indicates that dysbiosis of the intestinal microbiota is closely related to colorectal cancer (CRC). The roles for intestinal microorganisms that initiated and facilitated the CRC process are becoming increasingly clear. Hypothesis models have been proposed to illustrate the complex relationship between the intestinal microbiota and CRC. Recent studies have identified *Streptococcus bovis*, enterotoxigenic *Bacteroides fragilis*, *Fusobacterium nucleatum*, *Enterococcus faecalis*, *Escherichia coli*, and *Peptostreptococcus anaerobius* as CRC candidate pathogens. In this review, we summarized the mechanisms involved in microbiota-related colorectal carcinogenesis, including inflammation, pathogenic bacteria, and their virulence factors, genotoxins, oxidative stress, bacterial metabolites, and biofilm. We also described the clinical values of intestinal microbiota and novel strategies for preventing and treating CRC.

## Introduction

Colorectal cancer (CRC) is one of the most common cancers and is a major global health burden. CRC ranks third in terms of incidence and second in mortality worldwide, accounting for 1.8 million new cases and 881,000 deaths in 2018 (1). With its continued progression in western countries, the incidence of CRC is predicted to increase to 2.2 million new cases and 1.1 million deaths worldwide by 2030 (2). In China, over 376,000 new cases and 191,000 deaths are estimated to occur annually (3). CRC incidence and mortality have decreased steadily in recent decades among adults aged ≥65 years because of increased colonoscopy screenings; however, the opposite has occurred in adults younger than 50 years (4). In the United States from the mid-1980s through 2013, the colon cancer incidence increased by 2.4% annually in adults aged 20–29 years and by 1.0% annually in adults aged 30–39 years. It has also increased by 1.3% annually in adults aged 40–49 years and by 0.5% annually in adults aged 50–54 years since the mid-1990s (5, 6). This trend in younger adults, along with the continued burden in the overall population, is alarming; therefore, new strategies for early detection and prevention of CRC are urgently needed. As with many diseases, the CRC etiology is highly complex and involves both genetic and environmental factors (7). Evidence from twin and family studies indicates that only a small fraction of CRCs, including familial adenomatous polyposis (FAP), hereditary nonpolyposis colorectal cancer (HNPCC or Lynch syndrome), Peutz-Jeghers syndrome, and other more rare disorders, are genetically predisposed (8–10). In addition, most CRCs are sporadic or non-inherited (11). Environmental factors, such as western dietary habits, smoking, weight gain and obesity, diabetes, and heavy alcohol consumption, play major roles in causing sporadic CRC. Among environmental risk factors, the intestinal microbiota is an important contributor (12–15). Increasing evidence indicates that the intestinal microbiota plays a vital role in CRC initiation, progression, and metastasis (16, 17).

The human intestinal microbiota is composed of 10^13^ to 10^14^ microbes, encompasses ∼10 times more bacterial cells than human cells and contains > 100 times as many genes as in the human genome (18, 19). A healthy human intestinal microbiota plays a crucial role in harvesting energy (20), shaping the intestinal epithelium (21), protecting against pathogens (22), and maintaining immunity (23). Contrariwise, intestinal microbiota dysbiosis alters host physiological functions, leading to various diseases (24). Emerging studies on the relationship between the intestinal microbiota and CRC have analyzed the role of the intestinal microbiota in colorectal carcinogenesis. These studies have shown the differences in the intestinal microbiota compositions between patients with CRC and healthy individuals as well as which microbes are enriched or depleted in patients with CRC. Interestingly, microbiome alterations also occur with colorectal adenoma, the early stage of CRC. Thus, microbiome alterations might be used as biomarkers for early CRC detection. On the other hand, previous research findings suggest that modulating the intestinal microbiome may be a new strategy for CRC prevention and treatment. In this review, we summarized recent advances in understanding the associations between the intestinal microbiota and CRC based on evidence from animal and human studies, especially in terms of mechanisms. We also partly describe the clinical value of the intestinal microbiota and novel strategies for preventing and treating CRC.

## Intestinal Microbiota in CRC

In as early as the 1970s, animal experiments showed that intestinal microbial populations were crucial in mediating the carcinogenic effects of different compounds in intestine (25–27). For example, Wynder group used germ-free and conventional rats to study the effects of the intestinal microbiota on colonic sensitivity to the carcinogenic effect of 1,2-dimethylhydrazine. They found that only 20% of germ-free rats developed colonic tumors, whereas 93% of conventional rats developed multiple colonic tumors (26), and subcutaneously injecting azoxymethane increased the incidence and multiplicity of the colonic tumors in germ-free rats compared with that in conventional rats (27). Intestinal microbial dysbiosis could be observed within the intestines of mice with both spontaneous and chemically induced colon tumorigenesis. For example, *Apc* ^Min/+^ mice, a familial model of colonic tumor disease, spontaneously developed intestinal tumors due to a mutation in the adenomatous polyposis coli (*APC*) tumor-suppressor gene. The intestinal microbial diversity was reduced in C57BL/6J *Apc* ^Min/+^ mice compared with that in wild-type C57BL/6J mice (28). Additionally, *Apc* ^Min/+^ mice gavaged with feces from CRC patients exhibited enhanced intestinal adenoma progression (29).

High-throughput microbiome sequencing enables researchers to study microbial communities that colonize tumors as well as nontumor colonic sites and characterize individualized oncogenic microbiomes (13). Patients with CRC have shown reduced bacterial diversity and richness compared with those of healthy individuals (30). Additionally, although no unifying CRC-associated microbiota structure has yet been determined, accumulating human studies have shown that compared with matched microbiotas from healthy individuals, the intestinal microbiotas of CRC patients are structurally separate in both fecal (13, 31–35) and mucosal (12, 30, 36) samples. In addition, emerging studies have demonstrated the role of fungi in colorectal tumorigenesis. A previous study identified that fungal composition were different in tissue biopsies of 27 subjects with colorectal adenoma and adjacent tissues (37). Consistently, Yu et al. found that unlike the observation with bacteria, the fungal alpha diversities were not significantly different between CRC patients and healthy individuals, but the compositions were obviously altered. The Basidiomycota:Ascomycota ratio was increased in CRC patients compared with healthy subjects. And class Malasseziomycetes was enriched in CRC while classes Saccharomycetes and Pneumocystidomycetes were depleted (15). Overall, the CRC microbiota exhibits dysbiosis, reflecting a different ecological microenvironment in patients with CRC.

Despite the variations in intestinal microbiota, several individual bacterial species have been associated with CRC (**Table 1**). *Streptococcus bovis* (*S. bovis*), a gram-positive cocci, is a reported risk factor for CRC (38–40). Enterotoxigenic *Bacteroides fragilis* (ETBF), a bacterium producing *B. fragilis* toxin (BFT), causes diarrhea and inflammatory bowel disease (IBD) (41–44). *Fusobacterium nucleatum* (*F. nucleatum*) is enriched in human colorectal adenomas and carcinomas (45, 57) and may contribute to disease progression from adenoma to cancer (46). In our recent study, *F. nucleatum* was significantly increased in patients with early-stage CRC (49). The presence of *F. nucleatum* in CRC tissues indicated a worse prognosis (47, 48). Some studies reported that *Enterococcus faecalis* (*E. faecalis*) was significantly higher in patients with CRC compared with that in healthy controls (31, 51). *E. faecalis* infection induces superoxide production, which damages DNA in epithelial cells (50, 52). Although *Escherichia coli* (*E. coli*) is a gut commensal bacterium, studies have reported higher levels of colonic colonization by mucosa-associated *E. coli* in CRC patients compared with that in healthy people (53, 54, 58). Yu et al. found *Peptostreptococcus anaerobius* (*P. anaerobius*) was significantly enriched in fecal and mucosal microbiotas from patients with CRC (55, 56). Notably, it is not one specific microorganism that is responsible for CRC, but a group of bacteria whose detrimental actions surpass those of the beneficial commensals. On the other hand, some bacteria, mostly probiotics such as the butyrate-producer, *Clostridium butyicum*, and lactate-producer such as *S. thermophilus*, are depleted in CRC patients. These bacteria may exert a protective effect against CRC.

**Table 1:** Colorectal cancer associated intestinal microorganism.

Microorganism	Phylum	Natural habitat	Characteristics in CRC	Effectors	References
*Streptococcus bovis*	Firmicutes	GI tract	Early sign for CRC		(38–40)
Enterotoxigenic *Bacteroides fragilis*	Bacteroidetes	GI tract	Detected in ~90% of CRC patients	BFT	(41–44)
*Fusobacterium nucleatum*	Fusobacteria	Oral cavity	Increased in CRC patients, indicate a worse prognosis	Adhesin FadA, Fap2	(45–49)
*Enterococcus faecalis*	Firmicutes	GI tract	Increased in CRC patients	Production of superoxide	(31, 50–52)
*Escherichia coli*	Proteobacteria	GI tract	Increased in CRC patients	Colibactin	(53, 54)
*Peptostreptococcus anaerobius*	Firmicutes	GI tract	Increased in CRC patients	PCWBR2	(55, 56)

GI tract, gastrointestinal tract; BFT, B. fragilis toxin; FadA, Fusobacterium adhesin A; PCWBR2, putative cell wall binding repeat 2.

## Hypothesis Models Associated With Intestinal Microbiota and CRC

CRC occurrence is related to changes in the overall intestinal flora structure as well as infection with one or several specific bacteria. To better understand the relationship between intestinal microbiota and CRC, researchers raised a few hypotheses as follows:

### The Alpha-Bug Hypothesis

Sears and Pardoll proposed the “Alpha-bug” hypothesis based on previous work on ETBF carcinogenesis in *Apc* ^Min/+^ mice (59). ETBF rapidly induced the exclusive activation of signal transducer and activator of transcription-3 (STAT3) with colitis characterized by T helper 17 (Th17) responses, which might promote cancer in cooperation with the modified colonic epithelium (42). The Alpha-bug hypothesis integrates the single intestinal bacterium and microbiome community views of microbial carcinogenesis. Alpha-bugs not only induce tumors directly, but also remodel the bacterial community to one that promotes Alpha-bug induction of intestinal mucosal immune responses and intestinal epithelial cell (IEC) alterations resulting in cancer. Additionally, Alpha-bugs can enhance carcinogenesis by selectively “crowding out” of cancer-protective intestinal bacteria. Potential Alpha-bug candidates include ETBF, *S. bovis*, superoxide-producing *E. faecalis*, and *E. coli*.

### The Driver-Passenger Model

Next-generation sequencing technology has allowed us to explore the microbial composition of both healthy and diseased body sites. These studies have revealed that the intestinal microbiota associated with CRC contains bacterial species that differ in their temporal associations with developing tumors. Based on this, Tjalsma et al. first proposed a bacterial counterpart of the genetic “driver-passenger” model for CRC (60). This model classifies microbes into two categories. First, certain indigenous intestinal bacteria (termed driver bacteria) produce genotoxic substances to damage the epithelial cell DNA, thus initiating CRC. Second, tumor environmental alterations that favor proliferation of opportunistic bacteria (termed passenger bacteria), such as *Fusobacterium* spp., mediate colorectal tumorigenesis. Although the driver bacterial aspect of the driver-passenger model is related to the Alpha-bug hypothesis, it differs from the Alpha-bug model (61). The driver-passenger model highlights that although the driver bacteria initiate CRC, these bacteria will not always exist, and will be replaced by passenger bacteria as a loss of growth advantage, whereas the Alpha-bug hypothesis posits that driver bacteria persistently colonize developing tumors. Thus, these authors suggest that bacterial drivers and passengers have distinct temporal associations with CRC tissue. This model well explains the various results among different studies.

## Mechanisms in Colorectal Carcinogenesis

**Figure 1:**
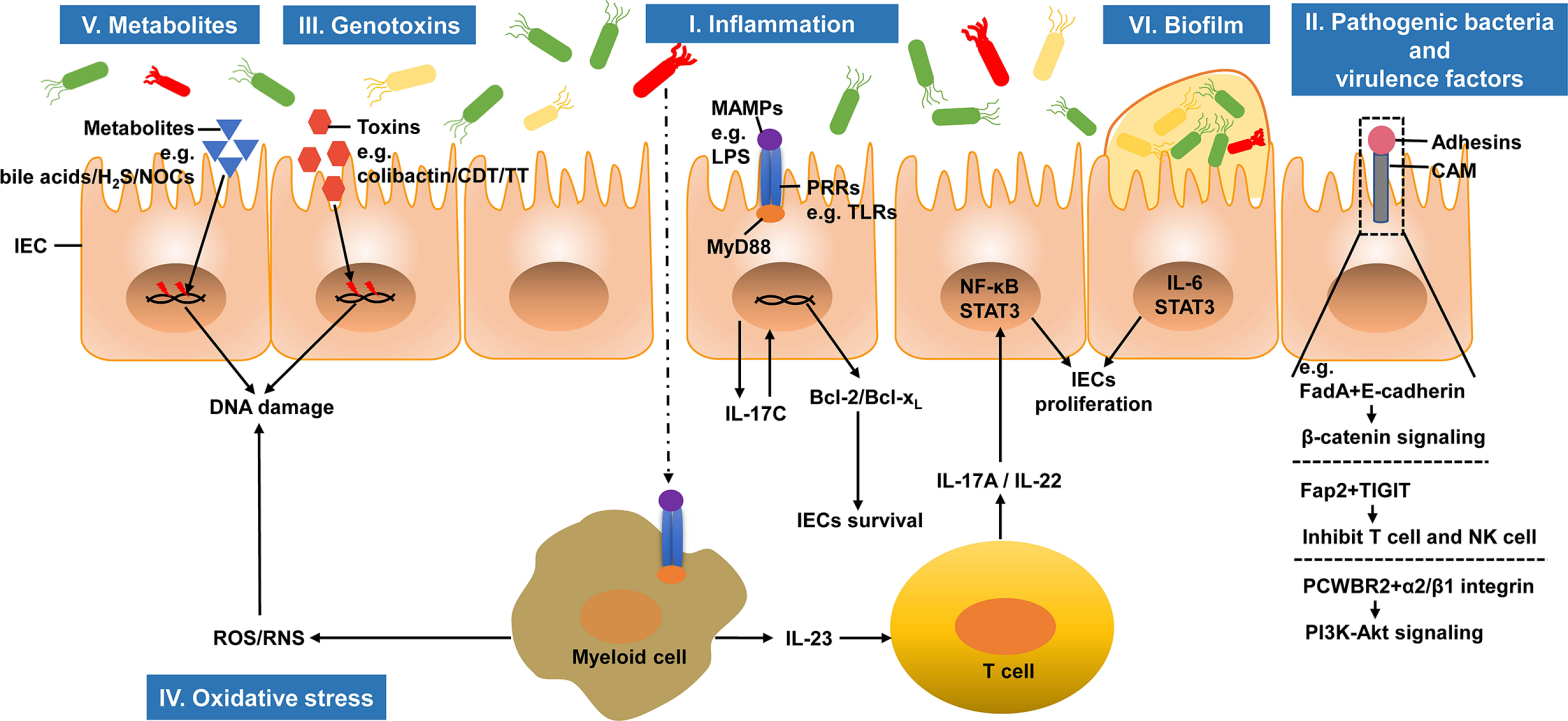
Microbiota-associated mechanisms in colorectal carcinogenesis. The intestinal microbiota can regulate the initiation and progression of CRC. I. The infiltration of commensal bacteria or their products activates tumor-associated myeloid cells and induces tumor promoting inflammation. II. Pathogenic bacteria and their virulence factors adhere to IECs and promote tumorigenesis. III. Genotoxins produced by bacteria induce DNA damages in IECs and initiate CRC development. IV. Under the stimulation of chronic inflammation, inflammatory cells can produce ROS and RNS, which in turn induce DNA damage. V. Several bacterial metabolites, including secondary bile acids, H_2_S and NOCs, can cause DNA damage, which promote CRC carcinogenesis. VI. Biofilm, microbial communities, promotes carcinogenesis through IL-6 and its downstream effector STAT3 activation. IEC, intestinal epithelial cell; H_2_S, hydrogen sulfide; NOCs, N-nitroso compounds; CDT, cytolethal distending toxin; TT, typhoid toxin; MAMP, microbe-associated molecular pattern; LPS, lipopolysaccharide; PRR, pattern recognition receptor; TLR, Toll-like receptor; MyD88, myeloid differentiation factor 88; NF-κB, nuclear factor-κB; STAT3, signal transducer and activator of transcription 3; CAM, cell adhesin molecule; FadA, *Fusobacterium* adhesin A; TIGIT, T-cell immunoglobulin and ITIM domain; PCWBR2, putative cell wall binding repeat2; ROS, reactive oxygen species; RNS, reactive nitrogen species.

Colorectal carcinogenesis is highly complex and involves genetic and environmental factors. Emerging studies suggest that several mechanisms, including inflammation, pathogenic bacteria, genotoxins, oxidative stress, metabolites, and biofilm, are closely linked to the intestinal microbiota. Here, we review the known microbiota-associated mechanisms in CRC carcinogenesis (**Figure 1**).

### Inflammation

The first connection between inflammation and cancer is attributed to Rudolf Virchow, who noted the presence of leukocytes in tumors in 1863 (62). Until the past decade that clear evidence has been obtained that inflammation acts as a critical factor in tumorigenesis. Chronic inflammation is widely accepted as a risk factor for CRC (63–65). Patients with IBD, including both ulcerative colitis (UC) and Crohn’s disease (CD), have higher risks of CRC. Previous meta-analyses summarized the 30-year cumulative risk of CRC, which is increased by up to 18.4% in UC patients (66) and 8.3% in CD patients (67).

The intestinal microbiota interacts closely with the host immune system. Bacterial stimulation of immune responses can cause continuous low-grade inflammation, resulting in tumorigenesis. Conversely, inflammation cannot induce CRC without the microbiota or bacteria-derived compounds (68). Therefore, increasing efforts have been made to understand tumor-elicited inflammation, which follows tumor development and can be detected in most solid malignancies (69).

Normally, the intestinal mucosal barrier segregates the intestinal microbiota from immune cells. The intestinal mucosal barrier is composed of a single layer of IECs joined by tight junctions (70). In both human and CRC mouse models, the intestinal mucosal barrier is highly permeable (71). Disrupting the intestinal mucosal barrier function *via* dextran sodium sulfate (DSS)-induced colitis leads to increased susceptibility to CRC (72). Specific ablation of matriptase, a membrane-anchored serine protease that strengthens the intestinal epithelial barrier by promoting tight junction formation, causes CRC development (73). All these suggest that transformed IECs fail to form an effective surface barrier, enabling commensal bacteria and their degradation products to invade the tumor stoma. Host recognizes the microbiota *via* various pattern recognition receptors [PRRs, such as Toll-like receptors (TLRs)], which control the inflammatory response to microorganism-associated molecular patterns, such as lipopolysaccharide (74). Invading commensal bacteria and their components engage TLRs on tumor-infiltrating myeloid cells following activation of myeloid differentiation factor 88 (MyD88)-mediated production of inflammatory cytokines, most notably interleukin (IL)-23. IL-23 then activates IL-17A, IL-6, and IL-22 production (71, 75, 76), eventually promoting tumor cell proliferation by activating nuclear factor-κB (NF-κB) and STAT3 signaling pathway (77, 78). Moreover, commensal bacteria and their components also upregulate IL-17C in transformed IECs through TLR/MyD88 dependent signaling. IL-17C induces B-cell lymphoma-2 (Bcl-2) and Bcl-x_L_ expression in IECs in an autocrine manner to promote tumor cell survival and tumorigenesis (75).

*F. nucleatum* generates a pro-inflammatory environment that permits colorectal neoplasia progression by activating the NF-κB pathway and recruiting tumor-infiltrating immune cells in *Apc* ^Min/+^ mice (45). IL-17A is highly expressed when *F. nucleatum* is enriched in human CRC (79).

ETBF secretes a 21 kDa BFT that cleaves E-cadherin on host IECs, thus disrupting the colonic barrier (80). Infecting *Apc* ^Min/+^ mice with ETBF selectively induces STAT3 activation with CRC characterized by Th17 responses (42). ETBF also promotes colonic cancer by secreting particles that stimulate IECs to produce exosome-like nanoparticles containing elevated levels of chemokine C-C motif ligand 20 and prostaglandin E2, which are required for Th17 cell recruitment and proliferation of IL-17 signals to transformed IECs to support their growth and survival (81).

*P. anaerobius* can provoke a pro-inflammatory immune microenvironment to promote tumorigenesis. In *Apc* ^Min/+^ mice, *P. anaerobius* broadly induces pro-inflammatory cytokine expression, which in turn recruits a series of tumor-infiltrating immune cells, especially immunosuppressive myeloid-derived suppressor cells, tumor-associated macrophages, and granulocytic tumor-associated neutrophils, to promote tumor progression (56).

### Pathogenic Bacteria and Their Virulence Factors

Several candidate pathogenic bacteria play vital roles in colorectal carcinogenesis by attaching to the mucosal surface. Bacterial adherence is often a prerequisite step to tumor promotion. *F. nucleatum*, an oral commensal bacterium, acts at the early step of colorectal carcinogenesis. Researchers demonstrated that *F. nucleatum* adheres to and induces CRC through its unique *Fusobacterium* adhesin A (FadA), which selectively binds to E-cadherin and activates the β-catenin signaling pathway, thus inducing oncogenic and inflammatory responses (82). Additionally, *F. nucleatum* inhibits T cell activation and natural killer cell cytotoxicity through its surface adhesin, Fap2, which binds to the human immune inhibitory receptor T-cell immunoglobulin and ITIM domain (83). A recent study showed that Fap2-dependent invasion induced secretion of the proinflammatory cytokines, IL-8, and CXCL1, which promoted CRC cell migration (84). *F. nucleatum* also modulates autophagy in IECs by activating regulatory microRNAs (85, 86). *P. anaerobius* normally resides in the oral cavity and gut. Yu et al. found that *P. anaerobius* promoted CRC development in *Apc* ^Min/+^ mice *via* its surface protein, putative cell wall binding repeat 2 (PCWBR2). PCWBR2 directly binds to intestinal epithelial cell receptor integrin α2/β1 to initiate an oncogenic PI3K-Akt signaling pathway, which promotes tumor cell proliferation (56). *S. bovis*, occasionally presents in the human gastrointestinal tract flora and is increased in patients with CRC (87, 88). Its role in CRC development is likely inflammation-driven carcinogenesis *via*, but not limited to, IL-1, cyclooxygenase-2 (COX-2), and IL-8 (89, 90). *Salmonella* infection in human can be chronic and increase the risk of cancer. *Salmonella* promotes colonic tumorigenesis dependent on its protein AvrA, which can activate both the Wnt/β-catenin and STAT3 signaling pathways in colonic tumor cells (91–93).

### Genotoxins

Bacterially produced genotoxins are related to colonic carcinogenesis because of their DNA-damaging effects. *E. coli* harbors the genomic island, *polyketide synthase* (*pks*), which codes for production of the polyketide-peptide genotoxin, colibactin (94, 95). Cultured mammalian epithelial cells exposed to *pks+* *E. coli* exhibited transient DNA damage (94). In a xenograft model, researchers found that colibactin promoted cell senescence, followed by hepatocyte growth factor production and enhanced tumor cell proliferation (96). *Campylobacter jejuni* produces a genotoxin, cytolethal distending toxin, which causes double-stranded DNA breaks and promotes colorectal tumorigenesis (97). *Salmonella* also produces a genotoxin, typhoid toxin, which damages DNA *via* the PI3K pathway in colonic epithelial cells (98).

### Oxidative Stress

Oxidative stress is an imbalance between production of pro-oxidative molecules (e.g., reactive oxygen species (ROS) and reactive nitrogen species (RNS) and the effectiveness of anti-oxidative defenses. Oxidative stress is common in chronic inflammation caused by the intestinal microbiota. Under the stimulation of chronic inflammation, inflammatory cells produce many ROS and RNS, which can induce DNA damage and further activate oncogenes or inactivate tumor-suppressor genes, thus increasing CRC development. The gut microbiota can also directly produce ROS. *E. faecalis* infection in macrophages induces superoxide production, which damages DNA in epithelial cells *via* a bystander effect (50, 99). *In vitro* and *in vivo* studies demonstrated that *E. faecalis* can produce hydroxyl radicals (100, 101), which are powerful mutagens that cause DNA breaks, point mutations and protein-DNA crosslinking, thus contributing to chromosomal instability and CRC risk (102). *P. anaerobius* activates TLR2/TLR4 on IECs and boosts intracellular ROS levels, which promotes cholesterol synthesis and cell proliferation (55). *E. coli* and BFT of ETBF also promote ROS production by colonic epithelial cells (103, 104).

### Diet and Bacteria Metabolites

According to a recent study, 38.3% of CRC cases were related to poor diets with intake of food low in whole grains, low in dairy products and high in red and processed meats (105). Additionally, obesity, which increases CRC risk of by 19%, and being overweight have been recognized as significant risk factors for CRC (106). A report found that each 5-kg/m^2^ increase in body mass index is associated with a 5% increase in CRC risk (6). In a sense, these make CRC a somewhat preventable disease. Tilg et al. previously reviewed this topic, and they highlighted that the microbiota could indeed reflect a ‘‘missing link’’ in the close interaction between dietary factors and CRC (107). Diet affects CRC, partly by modulating the intestinal microbiome composition and diversity. For example, diets high in animal protein and fat yielded enterotypes dominated by *Bacteroides*, whereas diets high in carbohydrates yielded enterotypes dominated by *Prevotella* (108, 109). As a mediator between the diet and the host, the intestinal microbiota plays a considerable role in host metabolism. Undigested dietary components (e.g., fructo-oligosaccharides) reach the large intestine, and intestinal microbes ferment the host products (e.g., bile acids). Organic acids, particularly the three short-chain fatty acids (SCFAs) acetate, butyrate, and propionate, are the predominant fermentation products in healthy adults who consume balanced diets (7, 20). However, with unbalanced dietary patterns, microbial metabolism also generates pro-carcinogenic chemicals such as secondary bile acids, N-nitroso compounds (NOCs), and hydrogen sulfide (H_2_S) (110).

SCFAs are a major class of metabolites produced *via* microbial metabolism of dietary components. Although acetate, butyrate and propionate have health-promoting effects, butyrate is the most potent with respect to cancer protection. Butyrate, produced predominantly by *Firmicutes via* fermentation of dietary fiber and resistant starches, is the chief energy source for colonocytes and regulates epithelial proliferation. Butyrate can inhibit histone deacetylase activity in colonocytes and immune cells, consequently downregulating proinflammatory cytokines and inducing apoptosis in CRC cells (111, 112). SCFAs (especially butyrate) can significantly lower fecal pH in the colon, thereby inhibiting pathogenic bacterial proliferation and DNA damage, and enhancing apoptosis and preventing cancer cell proliferation (113). In addition, butyrate and propionate shape the mucosal immune system by regulating colonic regulatory T-cell differentiation (42, 112, 114). Extracellular SCFAs also participate in this process by interacting with host cell surface receptors such as G protein-coupled receptors (GPCRs), GPR41, GPR43, and GPR109A (115–117). In turn, reduced SCFA levels are linked to a high CRC risk, such as in patients with advanced colorectal adenoma (118).

Bile acid metabolism is another main type of microbial metabolism. Primary bile acids are produced in the liver from cholesterol and metabolized to secondary forms by intestinal bacteria. Increasing evidence has shown that fat may affect CRC risk *via* its role in bile acid metabolism in the host and intestinal microbiota. High-fat diets lead to increased secondary fecal bile acid concentrations in populations with a CRC risk (119, 120). In a long-term diet study, mice fed a western-style diet high in fat developed significantly more colonic tumors than did mice on a control diet, correlating with higher cell proliferation in colonic crypts, impaired bile acid transport, and altered activity of the farnesoid X receptor (FXR), a nuclear bile acid receptor. These results suggest that western-style diets increase cancer risk *via* FXR inactivation, leading to bile acid deregulation and increased colonocyte proliferation (121). In a dietary intervention trial, healthy African Americans consuming a high-fat, low-fiber diet had more fecal bile acids than did healthy rural Africans who consumed a low-fat, high-fiber diet. However, when these dietary patterns were swapped, this phenomenon changed accordingly (122). Secondary bile acids have been shown to be genotoxic *via* oxidative stress from ROS generation causing oxidative DNA damage (123).

High protein intake increases detrimental metabolites in the colon, such as NOCs and H_2_S. NOCs are positively correlated with CRC in European populations and can promote cancer and exert carcinogenic effects *via* DNA alkylation (124, 125). Sulfate-reducing bacteria may use methionine and cysteine as substrates, leading to H_2_S generation. A study showed that sulfate-reducing bacterial abundance was increased in the stools of CRC patients compared with those of healthy individuals (126). H_2_S can stimulate CRC progression by inhibiting butyrate oxidation and inducing breakdown of the gut barrier. H_2_S can also induce DNA damage *via* ROS (7, 127).

### Biofilm

Biofilm is an emerging concept in studying the relationship between the intestinal microbiota and CRC. Biofilms, aggregations of microbial communities encased in a polymeric matrix, invade the colonic mucosal layer and come into direct contact with mucosal epithelial cells. Dejea et al. reported that invasive polymicrobial bacterial biofilms were detected in most right-sided tumors (89%) but in only 12% of left-sided tumors and were accompanied by diminished IEC E-cadherin, increased epithelial permeability, and enhanced IL-6 and STAT3 activation. The IL-6 family and their downstream effector, STAT3, promote CRC through increased epithelial proliferation and diminished apoptosis. Consequently, Dejea et al. proposed a model that biofilm formation enhanced colonic epithelial permeability, which facilitates bacterial antigen translocation and promotes pro-carcinogenic tissue inflammation (128, 129).

## Clinical Value of the Microbiota

Studies exploring CRC mechanisms share the ultimate goal of better CRC prevention and treatment. Studies on the metagenomic landscape of the CRC microbiota have enabled selecting useful biomarkers, and investigations of microbiota-related mechanisms can help develop effective strategies for CRC prevention and treatment. Since this part is not the focus of this review, we kindly refer to the previous reviews for details (130, 131).

### Biomarkers for CRC Screening and Prognosis

Biomarkers represent a major translational application of the microbiota. Previous studies have shown that microbiota-related biomarkers may be used for screening and as prognostic tools for CRC treatment (**Table 2**).

**Table 2:** Intestinal microbiota biomarkers for colorectal cancer screening and prognosis.

Category	Matrix	Cohort	Study method	Candidate biomarker(s)	AUC	Reference
**Screening**	Human feces	120 CRC, 172 healthy controls	16S sequencing	FIT with 23 bacterial markers	0.95	(132)
	Human feces	83 CRC, 10 healthy controls	Digital PCR	FIT with fecal microbiome	0.98	(133)
	Human feces	39 CRC, 66 healthy controls	qPCR	*F. nucleatum*	0.737	(134)
	Human feces	367 CRC, 258 healthy controls	qPCR	Combination of two microbial ratios (*F. nucleatum* to *Bifidobacterium* and *F. nucleatum* to *Faecalibacterium prausnitzii*)	0.943	(135)
	Human feces	104 CRC, 102 healthy controls	qPCR	FIT with *F. nucleatum*	0.95	(136)
	Human oral swabs	25 CRC, 45 healthy controls	16S sequencing	Panel of 16 oral markers	0.905	(137)
**Prognosis**	Human cancer tissues	1069 CRC	qPCR	*F. nucleatum*		(47)

AUC, area under the receiver operating characteristic curve; FIT, fecal immunochemical test; qPCR, quantitative PCR; F. nucleatum, Fusobacterium nucleatum.

Several studies found that alterations in the fecal microbiomes of patients with CRC also occurred in patients with colorectal adenoma, which is recognized as a precursor of most CRCs. Hence, these might be used to screen individuals at risk for CRC, who can be treated in time with excellent clinical outcomes. An effective screening biomarker leading to early detection would substantially reduce CRC-related mortality. The 5-year survival rate of patients with localized CRC is 90%, while that of patients with distant metastatic diseases is only 14% (138). In addition to classic invasive endoscopic approaches, several early noninvasive CRC screening tools, such as fecal immunohistochemical testing (FIT), have been widely used because of their effects on reducing both CRC incidence and mortality. However, these techniques have been criticized for their relatively low sensitivity. FIT has only 79% sensitivity for detecting CRC and 25%–27% sensitivity for detecting advanced colorectal adenomas (139, 140). Therefore, efficient, safe, affordable, and noninvasive screening tools with high sensitivity for CRC are needed, and accumulating metagenomic CRC datasets may enable this. Some studies have demonstrated the potential for combining fecal microbiome data with FIT to improve CRC detection (132, 133). For example, fecal *F. nucleatum* stands out as highly valuable among several candidate biomarkers (134–136). Adding fecal *F. nucleatum* quantitation to FIT increases the area under the receiver operating characteristic curve from 0.86 to 0.95 (136). Some groups also try to find oral biomarkers associated with CRC detection, such as *Streptococcus* and *Prevotellas* pp. (137). In this study, researchers developed an oral microbiota-based classifier that distinguished patients with CRC and adenomas from healthy individuals. Screening the fecal metabolome is another promising non-invasive procedure for obtaining a unique metabolic fingerprint to diagnose CRC, although few studies with different metabolomic methods have shown the diagnostic potential of metabolites such as SCFAs (141). Apart from the potential for CRC screening, bacterial biomarkers may also serve as prognostic biomarkers. For example, Mima et al. found that larger amounts of *F. nucleatum* in CRC tissue were associated with worse clinical outcomes, including shorter survival times and a worse prognosis (47).

Finally, many studies have explored associations between microbial markers and CRC, but to date, no universal microbial marker is defined for CRC detection. The complexity of the microbiome presents various challenges. First, the high variability of the intestinal microbiota compositions among different populations owing to sex, age, diet, drug use, genetic background, and geographic location make identifying a universal microbial marker impossible. Thus, validating CRC screening markers for different populations and identifying core biomarkers that are robust across populations may be a possible solution in future studies. Second, limitations in techniques, such as different sample collection and storage methods and various analysis processes should be considered. Hence, standardized methods of sample collection, standardized analysis processes and unified quantitative standards for the microbial markers are needed. Therefore, scientists’ unremitting efforts are needed to overcome these scientific and technical challenges to allow better clinical translation.

### Microbiota Modulation for CRC Prevention and Treatment

Another translational application is microbiota modulation for CRC prevention and treatment. As described previously, the intestinal microbiota plays a major role in CRC *via* several mechanisms. Therefore, intestinal microbiota modulation, which aims to reverse established microbial dysbiosis, is a novel strategy for CRC prevention and treatment. Different strategies, such as dietary intervention, probiotics, prebiotics, and fecal microbiota transplantation (FMT), have been employed.

First, dietary factors are critical in CRC evolution. Dietary intervention is considered the most reasonable and economical approach to CRC treatment (142). Previous studies have shown the possibility of applying dietary strategies to modulate the intestinal microbiota. Populations consuming different diets have markedly different intestinal microbial compositions. Dietary intervention encourages the growth of certain bacterial strains that may convert indigestible dietary components into beneficial metabolites for the host. One systematic review of cohort studies showed that instead of western diets, adopting a healthy dietary pattern (high intake of fruits and vegetables, whole grain cereals, fish, white meats, and soy derivatives) decreased CRC risk (143). Consistently, in one study, 2-week food exchanges were performed between native Africans with low CRC rates and African Americans with high CRC rates. The African Americans consumed a high-fiber, low-fat diet, and the native Africans consumed a high-fat, low-fiber western diet. The dietary changes resulted in the African Americans exhibiting rapid and reciprocal changes in their intestinal microbiotas and mucosal biomarkers of CRC risk (122). Thus, higher fiber diets might be an effective method of treating CRC. Notably however, although short-term dietary intervention can rapidly reshape the intestinal microbiome, it cannot prevent CRC because once the original long-term diet is resumed, the intestinal microbiome returns to its previous composition (144). Moreover, obesity is positively correlated with CRC risk. Thus, reducing excessive dietary fat intake is extremely important for preventing CRC.

Second, the other ideal method for modulating the microbiota may be direct consumption of probiotics and/or prebiotics. The International Scientific Association for Probiotics and Prebiotics defines probiotics as “live microorganisms that, when administered in adequate amounts, confer a health benefit on the host” (145). Probiotics may function in CRC prevention and treatment by inactivating carcinogens or mutagens, modulating host immunity, inhibiting cell proliferation, and improving gut barrier function (131). Several chemical-induced animal model studies evidenced that administering probiotics exerted significant protective effects against CRC. *Faecalibacterium prausnitzii*, a potential probiotic, produces hydrophobic microbial anti-inflammatory molecules that can downregulate the NF-κB pathway in intestinal epithelial cells and prevent colitis in animal models (146). Treatment with a mixture of probiotics (*Lactobacillus plantarum*, *L. acidophilus*, and *Bifidobacterium longum*) in CRC patients increased the amount of cell junction proteins, thereby improving intestinal mucosal barrier integrity (147). Oral intake of *L. casei* reduced the atypia of colorectal tumors in patients who had undergone resection (148). One probiotic intervention study revealed that patients with CRC who received *B. lactis* Bl-04 and *L. acidophilus* NCFM had increased abundances of butyrate-producing bacteria, such as *Faecalibacterium* and *Clostridiales* spp., and decreased abundances of CRC-associated genera, including *Fusobacterium* and *Peptostreptococcus* (149). Prebiotics are nondigestible food ingredients that feed beneficial intestinal bacteria and improve host health. Synbiotics are the combination of prebiotics and probiotics. Numerous clinical trials have reported the effects of synbiotics on patients with CRC, including fewer postoperative infections and shorter hospital stays (150, 151). A synbiotic consisting of prebiotic inulin and the probiotics, *L. rhamnosus* GG (LGG) and *B. lactis* Bb12, reduced colorectal proliferation and improved epithelial barrier function in patients with histories of colonic polyps (152). Additionally, probiotic administration can ameliorate the adverse effects of chemotherapy and immunotherapy. Several studies have reported positive effects of probiotic use in CRC, including reduced diarrhea incidence, enhanced gut barrier integrity, and reduced inflammation (147, 153–155). For example, Osterlund et al. suggested that LGG supplementation might reduce the frequency of severe diarrhea and abdominal discomfort in CRC patients receiving 5-fluorouracil (5-FU) (156). Probiotics can also be useful in radiation therapy. A previous study found that probiotics could repair radiation-induced injuries (157). Hence, probiotics may be a potential complement in CRC prevention and treatment.

Third, FMT is an emerging biotherapeutic because of the increased understanding of how an altered intestinal microbiota affects diseases (158). Transferring stool transplants from healthy donors to patients believed to harbor a disease-inducing altered microbiota enables FMT to bring a healthy, disease-free microbiome into the patient’s gastrointestinal tract, which then restores eubiosis and may ameliorate various gastrointestinal disorders, including *C. difficile* infection (CDI), IBD and irritable bowel syndrome (159). Compared with other modulatory strategies, FMT has its own advantages and is the most direct method of shaping the microbiome with the most evidence of clinical efficacy. Currently, FMT is an established treatment for recurrent and refractory CDI, with cure rates of 80%–90% (160). Although its application in clinical CRC treatment is unexplored, a recent mouse study showed that introducing of fecal transplants from wild to laboratory mice promoted host fitness and improved resistance against DSS/azoxymethane (AOM)-induced colorectal tumorigenesis (161). Thus, FMT may be a novel CRC treatment strategy. Furthermore, modulating the intestinal microbiome *via* FMT may abrogate refractory colitis as an adverse effect of immunotherapy. Wang et al. reported the first human cases of immune checkpoint inhibitor-associated colitis successfully treated with FMT, with gut microbial reconstitution correlating with complete resolution of colitis for up to 53 days after one dose and 78 days after two doses (162).

In a word, modulating the intestinal microbiota in various ways may improve CRC prevention and treatment. Previous efforts to elucidate oncogenic mechanisms yielded an unprecedented opportunity to explore new strategies for diagnosing and treating CRC, with promising results. However, these strategies have controversies and challenges. For example, although dietary interventions (such as high-fiber intake) may potentially prevent CRC, more clinical and nutritional studies are needed to establish the most appropriate conditions for both dosage and duration (6). Further, not all probiotics are useful or work the same, and their benefit depends on the strain, dosage, intervention duration, and intestinal transit time. The safety of probiotics is also controversial, and some less characterized probiotics can alter the intestinal barrier. Therefore, further investigations are needed to identify safe and effective probiotics for CRC therapy and standardize the application and regulatory frameworks. Orally administered probiotics face an important technical problem that could minimize their efficacy. When they reach the colon, probiotics often lose their viability. Thus, new techniques, such as microencapsulation, must be developed to ensure their viability. Of course, some studies have already reported promising results for these techniques in animal models. More preclinical and clinical studies are needed to elucidate their availability in humans (163). Although FMT applications in treating recurrent and refractory CDI are highly successful, in CRC, they have only been used in animal models, and their clinical use in other diseases requires more supporting data from controlled trials. Currently, FMT is considered as a safe method with few adverse effects, but the long-term outcomes remain unclear. Furthermore, much information regarding the human intestines remains unclear, such as intestinal viral and fungal compositions and intestinal bacterial functions. Hence, disease transmission between the donor and recipient remains a risk. Accordingly, future research should focus on identifying the intestinal microbiota, defining its function, and developing defined microbial communities as alternatives to whole feces transplantation. A sound post-FMT follow-up system must be established to monitor the clinical efficacy and long-term adverse events. Additionally, FMT lacks a unified regulatory framework, and different countries have different regulations. Therefore, formalization of regulatory frameworks becomes other essential issue (164).

## Conclusion

The intestinal microbiota, often referred to as a “forgotten organ”, is gradually unraveling its mysterious veil. Numerous studies suggest that the intestinal microbiota is crucial in the CRC pathogenesis. Studies on CRC mechanisms have provided many new ideas for CRC prevention and treatment. However, because of individual variations, tumor stages, and cross-species translation, many challenges remain to be overcome in clinical practice. Continuous efforts in preclinical and clinical research are needed to better understand the links between the intestinal microbiota and CRC. In the near future, the intestinal microbiota will likely become a powerful weapon in fighting CRC.

## Author Contributions

YC, ZL, and LL discussed the contents, wrote, reviewed, and edited the manuscript. All authors contributed to the article and approved the submitted version.

## Funding

This present work was funded by the grants of the National S&T Major Project of China (2018YFC2000500), and the National Natural Science Foundation of China (81771724, 31700800, 81790631), the Foundation of China’s State Key Laboratory for Diagnosis and Treatment of Infectious Diseases.

## Conflict of Interest

The authors declare that the research was conducted in the absence of any commercial or financial relationships that could be construed as a potential conflict of interest.
